# Integrated metabolomic and transcriptomic analysis reveals the biosynthesis mechanism of dihydrochalcones in sweet tea (*Lithocarpus litseifolius*)

**DOI:** 10.3389/fpls.2025.1629266

**Published:** 2025-08-04

**Authors:** Shaojun Ling, Qiongqiong Lin, Biaofeng Zhou, Yiye Liang, Wenji Luo, Zhao Shen, Jingshu Wang, Jingwei Niu, Liangjing Qiao, Baosheng Wang, Hui Liu

**Affiliations:** ^1^ Guangdong Provincial Key Laboratory of Applied Botany, South China Botanical Garden, Chinese Academy of Sciences, Guangzhou, China; ^2^ State Key Laboratory of Plant Diversity and Specialty Crops, South China Botanical Garden, Chinese Academy of Sciences, Guangzhou, China; ^3^ South China National Botanical Garden, Guangzhou, China

**Keywords:** biosynthetic pathway, candidate genes, transcription factors, comparative genomics, biosynthetic gene clusters

## Abstract

The demand for plant-based, low-calorie natural sweeteners is increasing. Four dihydrochalcones (DHCs), namely phloretin, phlorizin, trilobatin, and sieboldin, have been identified in the leaves of *Lithocarpus litseifolius*. These compounds serve as natural flavor sweeteners with potential health-promoting effects. However, the biosynthetic pathways of these DHCs are not yet fully understood. In this study, the content of four DHCs was quantified using LC-MS/MS across five developmental stages (S1–S5) of *L. litseifolius* leaves. Our results revealed an accumulation pattern where DHC levels peaked at stage S3, followed by a sharp decrease at stages S4 and S5, with the exception of sieboldin, which maintained high levels. We elucidated the complete biosynthetic pathway of DHCs, involving 82 candidate enzyme-encoding genes, including five *PALs*, three *C4Hs*, 13 *4CL*s, 18 *CDBRs*, five *CHSs*, 14 *P2’GTs*, 12 *P4’GTs*, nine *F3’Hs*, and three *CH3H*s, and found that either tandem duplication or proximal duplication may have contributed to the expansion of key genes such as *CDBR*, *P2’GT*, and *P4’GT*. Furthermore, we reconstructed 11 regulatory networks of DHCs, two modules were positively related to the contents of phloretin, phlorizin, and trilobatin (r = 0.54–0.69, *P* < 0.05), while two other modules were associated with sieboldin accumulation (r = 0.59–0.74, *P* < 0.05). We also identified *MYB*, *WD40*-like, *WRKY*, and *bHLH* transcription factors as potential regulators in the biosynthesis of four DHCs. We found two biosynthetic gene clusters of DHCs, including nine and four genes encoding *CDBR* and *P2’GT*, respectively. Syntenic and phylogenetic analyses revealed that these two BGCs may have experienced independent evolutionary processes within the Fagaceae family. Our study provides a theoretical foundation for the resource development and utilization of sweet tea. It also paves the way for the development of high-quality natural sweeteners.

## 1 Introduction

Obesity, a global public-health issue driven by sedentary lifestyles and over-consumption of calories, is a primary risk factor for diabetes. In response, the food and beverage industry has promoted non-sugar sweeteners like aspartame, sucralose, and saccharin to reduce calorie intake (Kroger et al., 2006). However, the long-term negative effects of artificial sweeteners on human health were well documented (Brown et al., 2010; Castro-Muñoz et al., 2022; Ray and Palui, 2025), thus fueling the demand for natural alternatives (Wang and Lin, 2025). Recent research has focused on the commercialization of plant-derived sweeteners, such as stevia and monk fruit, which are considered healthier and more sustainable alternatives (Kumar et al., 2021; Chowdhury et al., 2022; Debras et al., 2022). Yet their limitations, such as the bitter aftertaste of stevia and the supply constraints of monk fruit, highlight the need for novel compounds. Dihydrochalcones (DHCs), a flavonoid subclass, present a viable solution (Dwivedi, 2022). Approximately 256 DHCs have been identified across 30 plant families (Rivière, 2016), and compounds like phloretin and phlorizin have been shown to modulate obesity-related biological pathways (Ehrenkranz et al., 2005; Dugé de Bernonville et al., 2010; Szliszka et al., 2010). Notably, trilobatin and phlorizin are 300 times sweeter than sucrose, serving as stable sweeteners and bitterness blockers (Tomás-Barberán and Clifford, 2000; Wang et al., 2020; Lou et al., 2023). DHCs combine sweet taste with obesity-related pathway regulation, making them ideal natural sweeteners for the food industry.

DHCs were first isolated from *Malus* species, though their concentrations are extremely low (Gosch et al., 2010; Gutierrez et al., 2018). However, the content of DHCs in tender leaves of *Lithocarpus litseifolius* is several times higher than that in apple peels (Qin and Liu, 2003; Wang et al., 2022; Zheng et al., 2025). Compared with stevia and monk fruit, *Lithocarpus litseifolius* can grow new leaves quickly after picking and pruning, allowing large-scale acquisition of leaves, thus showing obvious resource advantages. Moreover, its sweetness has a slow onset and a slow dissipation, with extremely stable properties (Tan et al., 2022). This makes *L. litseifolius* leaves an inexpensive source for dihydrochalcone production. *Lithocarpus litseifolius*, commonly known as sweet tea, is a species in the genus *Lithocarpus* of the family Fagaceae, which is an evergreen tree widely distributed in southern China (Cheng et al., 2016). Historically, *L. litseifolius* has been used to prepare medicinal drinks, and has been developed into sweet tea due to its high content of DHCs (Shang et al., 2022). This beverage is non-toxic, high in sweetness, and low in calories, making it an effective option for diabetes prevention and management (Qin and Liu, 2003; Lou et al., 2023; Wang et al., 2022). However, the biosynthesis mechanisms of DHCs in sweet tea remain partially understood. Identifying key candidate genes and regulatory pathways is crucial for enabling metabolic engineering to enhance DHC production, thus accelerating their application in medicine, food, and healthcare products.

So far, the biosynthetic pathways of phloretin, phlorizin, and trilobatin have been proposed (Wang et al., 2022; Lou et al., 2023). Specifically, glycosyltransferases related to the synthesis of phlorizin and trilobatin in sweet tea have been identified (Zhang et al., 2023), providing a fundamental understanding of the complete metabolic pathway of DHCs. DHCs are considered essential precursors and intermediates in the flavonoid synthesis pathway (Gosch et al., 2009; Barreca et al., 2014), yet the factors driving the metabolic diversity of DHCs remain unclear. Gene duplication, especially in enzymes like acyltransferases, methyltransferases, and glycosyltransferases, drives metabolite diversification (Leong and Last, 2017; Louveau and Osbourn, 2019; Hansen et al., 2021). Additionally, the biosynthesis gene clusters (BGCs) serve as synergistic factors in metabolic diversification (Liu et al., 2020; Zhou and Liu, 2022; Wang et al., 2024), and the discovery of BGCs for plant metabolites has opened new avenues for rapidly obtaining the biosynthetic pathways of target products (Ji et al., 2024; Zhu et al., 2024). Notably, while recent genomic studies have shed light on DHCs pathways (Lou et al., 2023; Zhang et al., 2023), critical gaps still persist: (1) the transcriptional regulatory networks governing phlorizin/trilobatin synthesis remain unmapped; (2) the biosynthetic pathway of sieboldin, a key bitterness modulator, remains uncharacterized; and (3) genomic mining of BGCs and their possible evolutionary dynamics and functional diversification across *Lithocarpus* species remain unresolved.

In this study, we determined the content of four DHCs during the development of tender leaves of sweet tea using LC-MS/MS. After that, we integrated genomic, metabolomic, and transcriptomic data to elucidate the complete biosynthetic pathway of DHCs, identifying candidate genes and key transcription factors (TFs). Furthermore, we conducted genome mining to identify duplicated genes and potential BGCs. The inter-species syntenic analysis and phylogenetic reconstruction within the Fagaceae family, shed light on the organization and evolution of candidate genes in BGCs.

## 2 Materials and methods

### 2.1 Plant material and sampling

**Figure 1:**
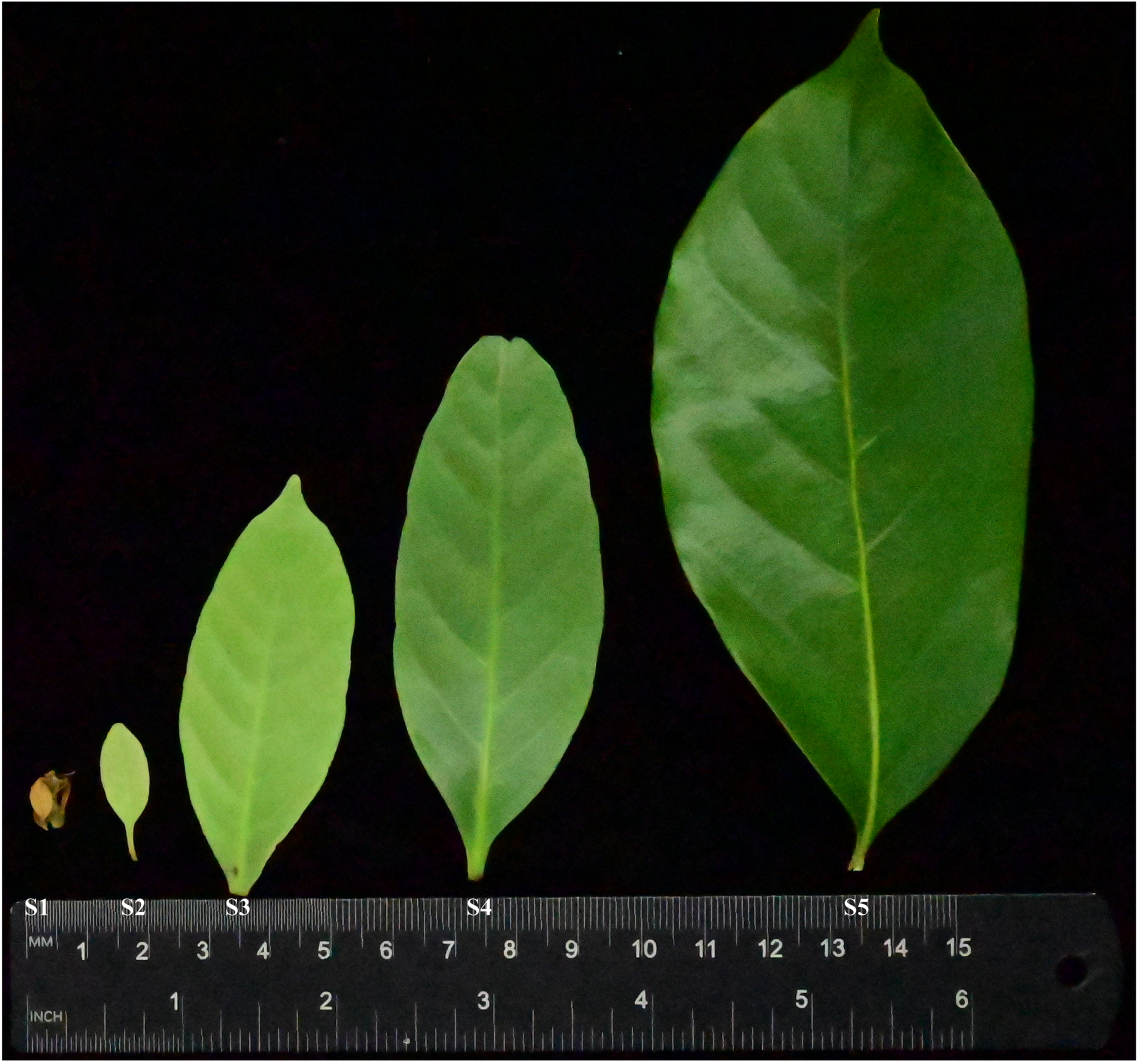
Leaf development stages (S1–S5) of sweet tea. S1 was described as newly-emerged juvenile leaves on February 03, 2023. S2–S5 were described as tender leaves collected at approximately 10-day intervals.

A healthy sweet tea tree located in the South China National Botanical Garden was selected for transcriptomic and metabolomic analysis. The genome of this tree has been assembled and annotated (Liu et al., 2023), providing crucial genomic resources for this study. Leaves at five distinct developmental stages (S1–S5, ranging from leaf bud to mature leaf; **Figure 1**) were collected at approximately 10-day intervals, with the first sampling conducted on February 3, 2023, for transcriptomic and metabolomic profiling.

### 2.2 Gene expression and metabolite profiling

Gene expression and metabolite patterns were investigated in leaves at five stages (S1–S5), with each stage including three biological replicates (**Figure 1**).

Transcriptomes were sequenced on the Illumina NovaSeq 6000 platform, generating approximately 90 Gb of 150 bp paired-end reads. High-quality reads were obtained by removing adapter sequences and filtering low-quality reads using Trimmomatic v0.40 (Bolger et al., 2014). To quantify gene expression levels, the clean reads were mapped to the sweet tea genome using HISAT2 v2.2.1 (Kim et al., 2019), and the counts for mapped reads were normalized by transcripts per million (TPM) using StringTie v2.2.1 (Pertea et al., 2015).

Quasi-targeted metabolomics was performed to analyze leaf metabolites. Briefly, leaves were subjected to liquid chromatography tandem mass spectrometry (LC-MS/MS), and multiple reaction monitoring (MRM) was applied to identify and quantify the metabolites. Metabolite quantification was conducted using daughter ions (Q3), while metabolite identification was carried out using parent ions (Q1), Q3, retention time (RT), declustering potential (DP), and collision energy (CE). The identified metabolites were annotated using Kyoto Encyclopedia of Genes and Genomes (KEGG, http://www.genome.jp/kegg/) and LIPID MAPS (http://www.lipidmaps.org/) databases. Tukey’s Honestly Significant Difference (HSD) test was performed to evaluate the statistical significance of content variations among samples. Pearson correlation between the gene expression level and the content of DHC metabolites was calculated using the cor function in R program.

### 2.3 Identification of candidate DHC biosynthesis genes

The complete DHC biosynthetic pathway involves nine well-characterized enzymes (Gosch et al., 2009; Gutierrez et al., 2018; Wang et al., 2022; Yahyaa et al., 2016), including phenylalanine ammonia-lyase (PAL), cinnamate 4-hydroxylase (C4H), 4-coumarate-CoA ligase (4CL), hydroxycinnamoyl-CoA double bond reductase (CDBR), chalcone synthase (CHS), phlorizin 2’-O-glycosyltransferase (P2’GT), phlorizin 4’-O-glycosyltransferase (P4’GT), flavonoid 3’-hydroxylase (F3’H), and chalone-3-hydroxylase (CH3H). To identify the DHC biosynthesis genes in sweet tea, we performed homologous alignment using BLASTP v2.13.0+ with the following settings: *E*-value < 1e-10, identity > 50%, and coverage > 85%, comparing the reference protein sequences of the above nine key enzymes obtained from UniProt (https://uniprot.org/) with the potential protein sequences of sweet tea.

### 2.4 Identification of duplicated genes

To characterize candidate genes involved in DHC biosynthesis, duplicated genes in sweet tea and 11 Fagaceae species were identified using “DupGen_finder-unique.pl” implemented in the DupGen_finder pipeline (Qiao et al., 2019), with *Nelumbo nucifera* (Li et al., 2020) serving as the outgroup. The identified duplicated genes were divided into five non-redundant categories, depending on their suspected origin: whole-genome duplication (WGD), tandem duplication (TD), proximal duplication (PD), transposed duplication (TRD), and dispersed duplication (DSD).

### 2.5 Weighted gene co-expression network analysis

To identify co-expression networks associated with DHCs biosynthesis, genes were grouped into modules with strongly covarying patterns across the five developmental stages of leaves using WGCNA v1.72-1 (Langfelder and Horvath, 2008), based on gene expression data with TPM > 0.5 in all samples. The modules were identified using the automatic network construction and module detection function (blockwiseModules implemented in WGCNA), with the following parameters: corType = “pearson’’, TOMType = “unsigned’’, minModuleSize = 200, mergeCutHeight = 0.15, deepSplit = 2, and maxBlockSize = 15,000. Pearson correlation between the eigengene values of modules and the content of DHC metabolites was calculated using the cor function in R program. The modules of the co-expression network were visualized using Cytoscape v3.10.0 (Shannon et al., 2003).

### 2.6 Biosynthetic gene clusters prediction and synteny analyses

Potential biosynthetic gene clusters (BGCs) associated with candidate genes involved in DHCs biosynthesis were predicted using plantiSMASH v2.0.0-beta (Kautsar et al., 2017, 2018). To analyze the local synteny of gene-pairs in potential BGCs, we generated microsynteny plots via the ‘synteny’ function in LAST (Kiełbasa et al., 2011). Whole genome synteny analysis between sweet tea and 11 Fagaceae species was performed using JCVI v1.2.7 (Tang et al., 2008).

### 2.7 Phylogenetic analyses

To investigate the conservation and evolution dynamics of predicted BGCs involved in DHC biosynthesis within the Fagaceae family, we constructed the phylogenetic relationship of sweet tea and 11 other Fagaceae species. First, a total of 3,498 single-copy orthologous genes from 12 species were identified using OrthoFinder v2.5.4 (Emms and Kelly, 2019). These genes were aligned using MAFFT v7.505 (Katoh and Standley, 2013), followed by the removal of gap positions using trimAl v1.4.rev15 (Capella-Gutiérrez et al., 2009). A maximum likelihood (ML) tree was constructed based on concatenated alignment using IQ-TREE v2.0.5 (Nguyen et al., 2015). We estimated the divergence time of the 12 Fagaceae species using MCMCTree in the PAML package v4.9j (Yang, 2007). Specifically, the split time of genus *Fagus* and other Fagaceae species at 82–81 million years ago (Ma; Grímsson et al., 2016), and the divergence time of *Castanopsis* and *Castanea* at 53–52 Ma was included to constrain the age of nodes (Wilf et al., 2019).

## 3 Results

### 3.1 Variation in DHC content during leaf development

**Figure 2:**
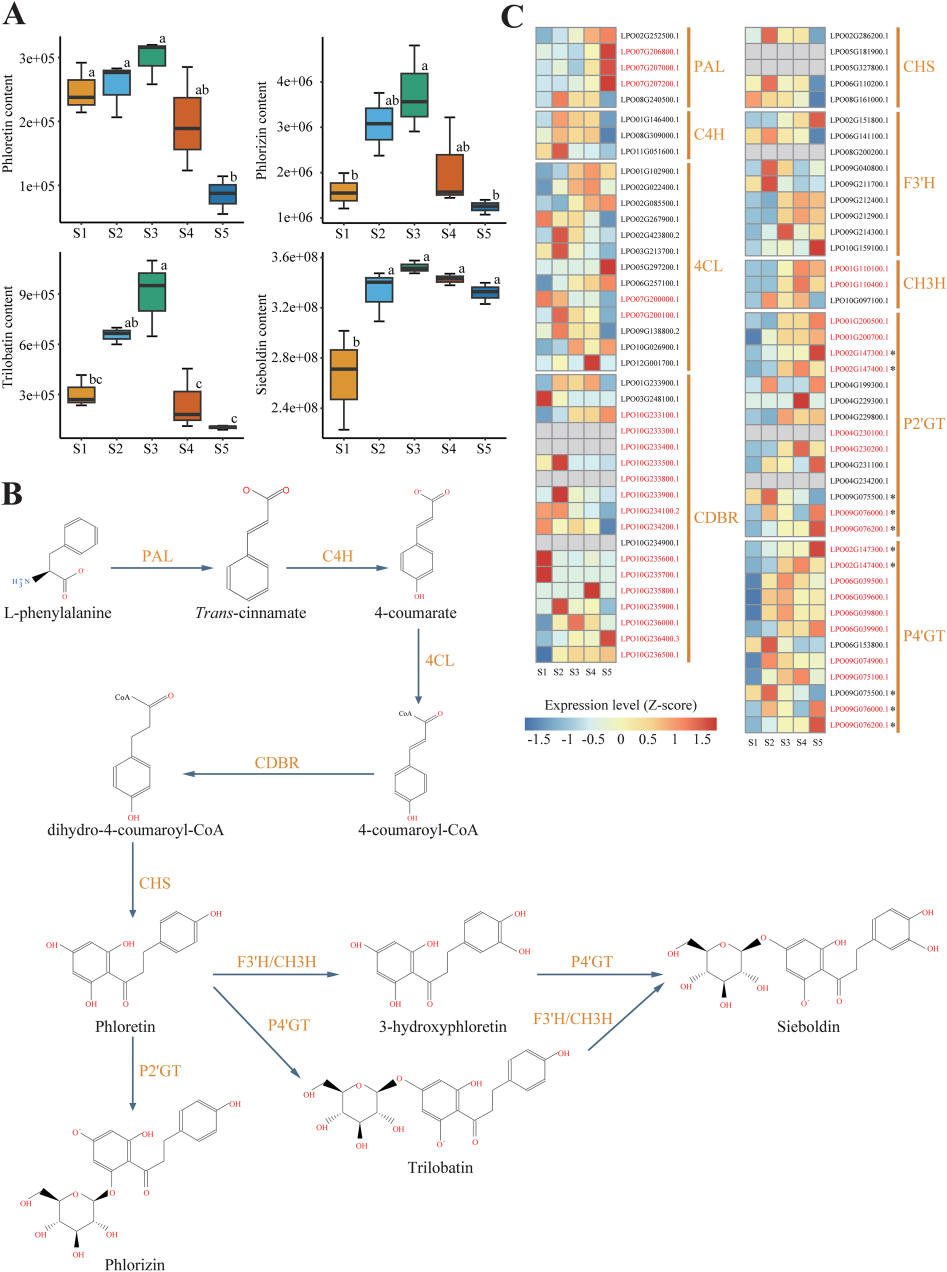
Biosynthesis of Dihydrochalcone (DHC) in sweet tea. **(A)** Boxplot analysis of the contents of DHCs, including phloretin, phlorizin, trilobatin, and sieboldin, in leaves at different developmental stages. Different letters denote statistically significant differences (*P* < 0.05; Tukey’s HSD test). **(B)** The biosynthetic pathway of DHC in sweet tea. *PAL*, phenylalanine ammonia-lyase; *C4H*, cinnamate 4-hydroxylase; *4CL*, 4-coumarate-CoA ligase; *CDBR*, hydroxycinnamoyl-CoA double bond reductase; *CHS*, chalcone synthase; *P2’GT*, phlorizin 2’-O-glycosyltransferase; *P4’GT*, phlorizin 4’-O-glycosyltransferase; *F3’H*, flavonoid 3’-hydroxylase; *CH3H*, chalone-3-hydroxylase. **(C)** Heatmap presenting the expression levels of genes encoding DHC biosynthesis enzyme in leaves at five different developmental stages. Gray boxes denote genes that show no expression at any of the developmental stages. Genes highlighted in red indicate those derived from tandem duplication (TD) or proximal duplication (PD). The asterisk represents five genes encoding *P2’GT* or *P4’GT*, which cannot be assigned to a specific one.

The relative metabolic contents of four DHCs (phloretin, phlorizin, trilobatin, and sieboldin) were measured in leaves of sweet tea at five developmental stages (S1-S5; **Figure 1**). The concentration of DHCs varied significantly across the five stages of leaf development. Specifically, leaves at stage S3, approximately 20 days after leaf bud flush, displayed the highest DHC content. The concentration of sieboldin remained consistently high throughout the period from stage S2 to stage S5, while the concentration of the other DHCs declined sharply after stage S3 (**Figure 2A**).

### 3.2 Candidate genes in DHCs biosynthesis

We reconstructed the complete biosynthetic pathway of DHCs based on the high-quality genome of sweet tea, and measured the expression levels of candidate enzyme-encoding genes involved in the pathway (**Figures 2B, C**). We identified 82 genes potentially participating in DHCs biosynthesis of sweet tea, including five *PALs*, three *C4Hs*, 13 *4CL*s, 18 *CDBRs*, five *CHSs*, 14 *P2’GTs*, 12 *P4’GTs*, nine *F3’Hs*, and three *CH3H*s (**Table 1**). Based on the gene expression levels associated with DHCs biosynthesis enzymes, we noted that several key genes showed high expression during the synthesis of four DHCs, including two *PALs*, two *C4Hs*, 10 *4CLs*, eight *CDBRs*, three *CHSs*, three *F3’Hs*, three *CH3Hs*, one *P2’GT*, and five *P4’GTs* (**Supplementary Figure S1**). For instance, the high expression levels of three *CHS* genes (*LPO02G286200.1*, *LPO06G110200.1*, and *LPO08G161000.1*) in stage S2 were positively related to higher concentrations of phloretin biosynthesis (r = 0.66–0.89, *P* < 0.05; **Supplementary Figure S1**). For phlorizin biosynthesis, one *P2’GT* gene (*LPO09G075500.1*) was highly expressed in stage S2 (r = 0.50, *P* < 0.05; **Supplementary Figure S1**).

**Table 1:** Number of enzyme-coding genes in dihydrochalcone (DHC) biosynthesis of sweet tea.

Species	*PAL*	*C4H*	*4CL*	*CDBR*	*CHS*	*P2’GT*	*F3’H*	*CH3H*	*P4’GT*
*Quercus mongolica*	4	3	14	21	2	17	7	3	24
*Quercus dentata*	4	2	13	21	3	17	6	2	21
*Quercus lobata*	4	3	14	20	4	15	9	2	23
*Quercus variabilis*	4	4	9	8	2	5	6	2	8
*Quercus acutissima*	4	0	11	12	4	6	7	2	9
*Quercus gilva*	6	1	10	15	2	9	9	1	3
*Lithocarpus litseifolius*	5	3	13	18	5	14	9	3	12
*Castanopsis tibetana*	5	3	15	27	4	10	9	1	23
*Castanopsis hystrix*	4	3	15	7	4	7	8	1	3
*Castanea mollissima*	5	3	17	3	2	18	4	2	19
*Castanea crenata*	5	2	9	16	2	10	6	0	9
*Fagus sylvatica*	7	4	12	24	4	22	7	4	22

*PAL*, phenylalanine ammonia-lyase; C4H, cinnamate 4-hydroxylase; *4CL*, 4-coumarate-CoA ligase; *CDBR*, hydroxycinnamoyl-CoA double bond reductase; *CHS*, chalcone synthase; *P2’GT*, phlorizin 2’-O-glycosyltransferase; *P4’GT*, phlorizin 4’-O-glycosyltransferase; *F3’H*, flavonoid 3’-hydroxylase; *CH3H*, chalone-3-hydroxylase.

The number of genes identified in each category enzyme-encoding gene within sweet tea closely resembled that of other Fagaceae species (**Table 1**). Moreover, five categories of duplicated genes detected in sweet tea exhibited strong similarity to their counterparts in other Fagaceae plants (**Supplementary Figure S2**). TD or PD increased the copy number of genes involved in DHC biosynthesis of sweet tea, specifically those encoding *CDBR*, *P2’GT*, and *P4’GT* enzymes (**Figure 2C**). Five genes encoding *P2’GT* or *P4’GT* could not be clearly assigned to a specific type. Four of these genes were derived from TD (*LPO02G147300.1* and *LPO02G147400.1*) or PD (*LPO09G076000.1* and *LPO09G076200.1*). In addition, the expression patterns of these four TD- or PD-derived genes were different from each other, which may indicate the functional divergence after gene duplication (**Figure 2C**).

### 3.3 Co-expression network and TFs involved in DHCs biosynthesis

**Figure 3:**
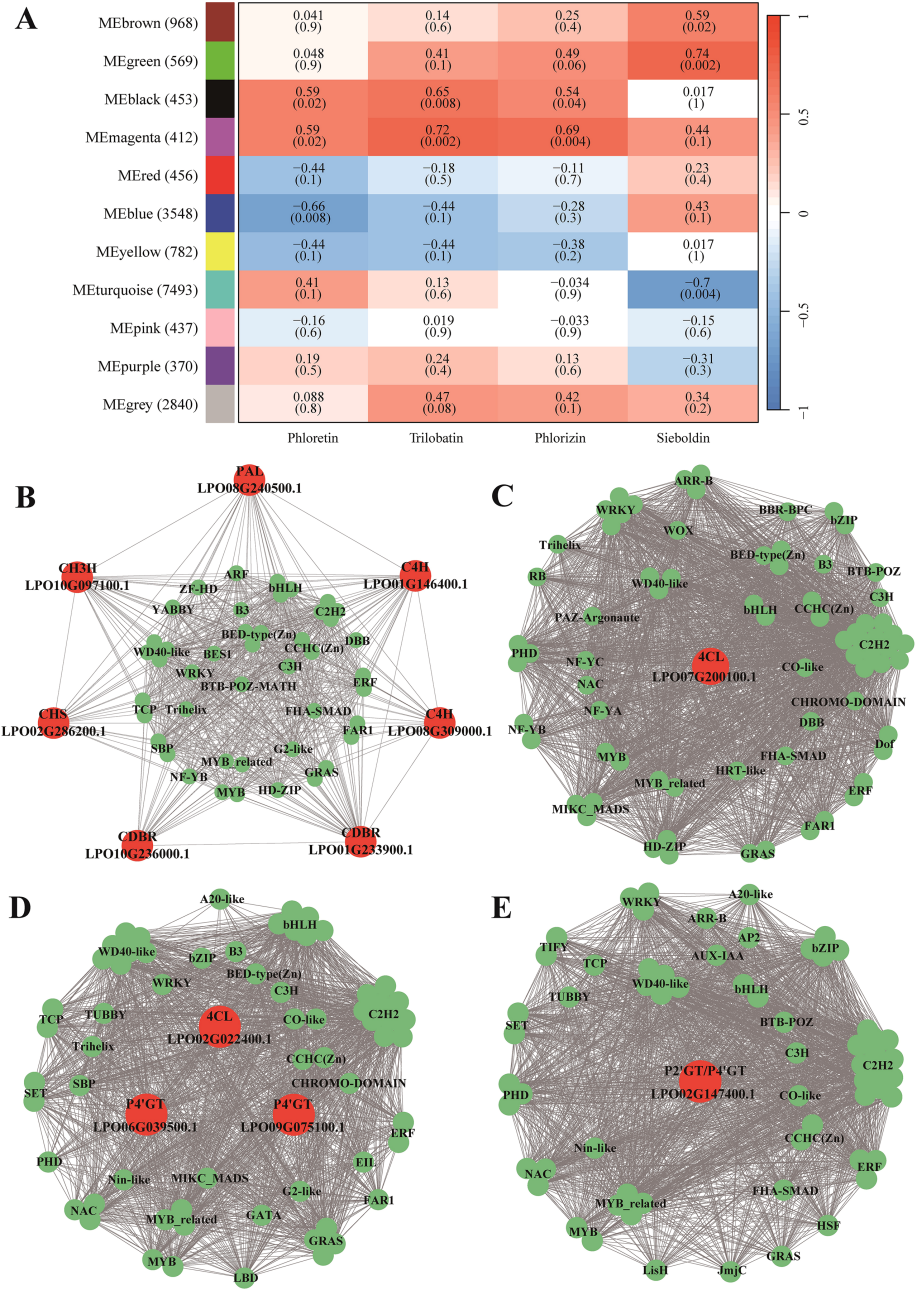
Transcriptional regulatory network governing DHC biosynthesis in sweet tea. **(A)** Correlations between the modules of the co-expression regulatory network and DHC metabolites. **(B–E)** Interactions between the candidate genes in DHC biosynthesis and transcription factors (TFs) within magenta **(B)**, black **(C)**, green **(D)**, and brown **(E)** modules. Red and green circles represent candidate genes and TFs in DHC biosynthesis, respectively.

To obtain insights into the regulation of DHCs biosynthesis, we constructed a gene co-expression network. A total of 11 modules, each containing 370–7,493 genes, were identified in the co-expression network (**Figure 3A**). Among the 11 modules, the magenta- and black-coded modules were positively correlated with the contents of phloretin, phlorizin, and trilobatin (r = 0.54–0.69, *P* < 0.05), while the green- and brown-coded modules were positively correlated with sieboldin content (r = 0.59–0.74, *P* < 0.05; **Figure 3A**). The magenta- and black-coded modules contained eight core genes in DHCs biosynthesis including one *PAL* (*LPO08G240500.1*), two *C4Hs* (*LPO08G309000.1* and *LPO01G146400.1*), two *CDBRs* (*LPO10G236000.1* and *LPO01G233900.1*), one *4CL* (*LPO07G200100.1*), one *CHS* (*LPO02G286200.1*), one *CH3H* (*LPO10G097100.1*) (**Figures 3B, C**), whereas the green- and brown-coded modules comprised five core DHC biosynthesis genes including one *4CL* (*LPO02G022400.1*), one *P2’GT*/*P4’GT* (*LPO02G147400.1*), and three *P4’GTs* (*LPO06G039500.1*, *LPO09G075100.1*, and *LPO02G147400.1*) (**Figures 3D, E**).

To identify transcription factors (TFs) involved in the biosynthesis pathway of DHCs, we selected those TFs that exhibited direct interactions with the candidate genes in the network modules. Specifically, we focused on TFs represented as the first-order neighbor nodes within the modules (**Figures 3B–E**). In the magenta-coded module, we found a total of 44 TFs belonging to 26 families involved in the regulation of seven genes, including one *PAL*, two *C4Hs*, two *CDBRs*, one *CHS*, and one *CH3H*. In the black-coded module, 73 TFs from 26 families were implicated in the regulation of a single gene, *4CL*. In the green-coded module, 63 TFs from 30 families participated in regulating two genes, one *4CL* and one *P4’GT*. In the brown-coded module, 63 TFs from 28 families were involved in the regulation of either *P2’GT* or *P4’GT* (*LPO02G147400.1*) (**Supplementary Table S1**).

### 3.4 Putative biosynthesis gene clusters involved in DHCs biosynthesis

**Figure 4:**
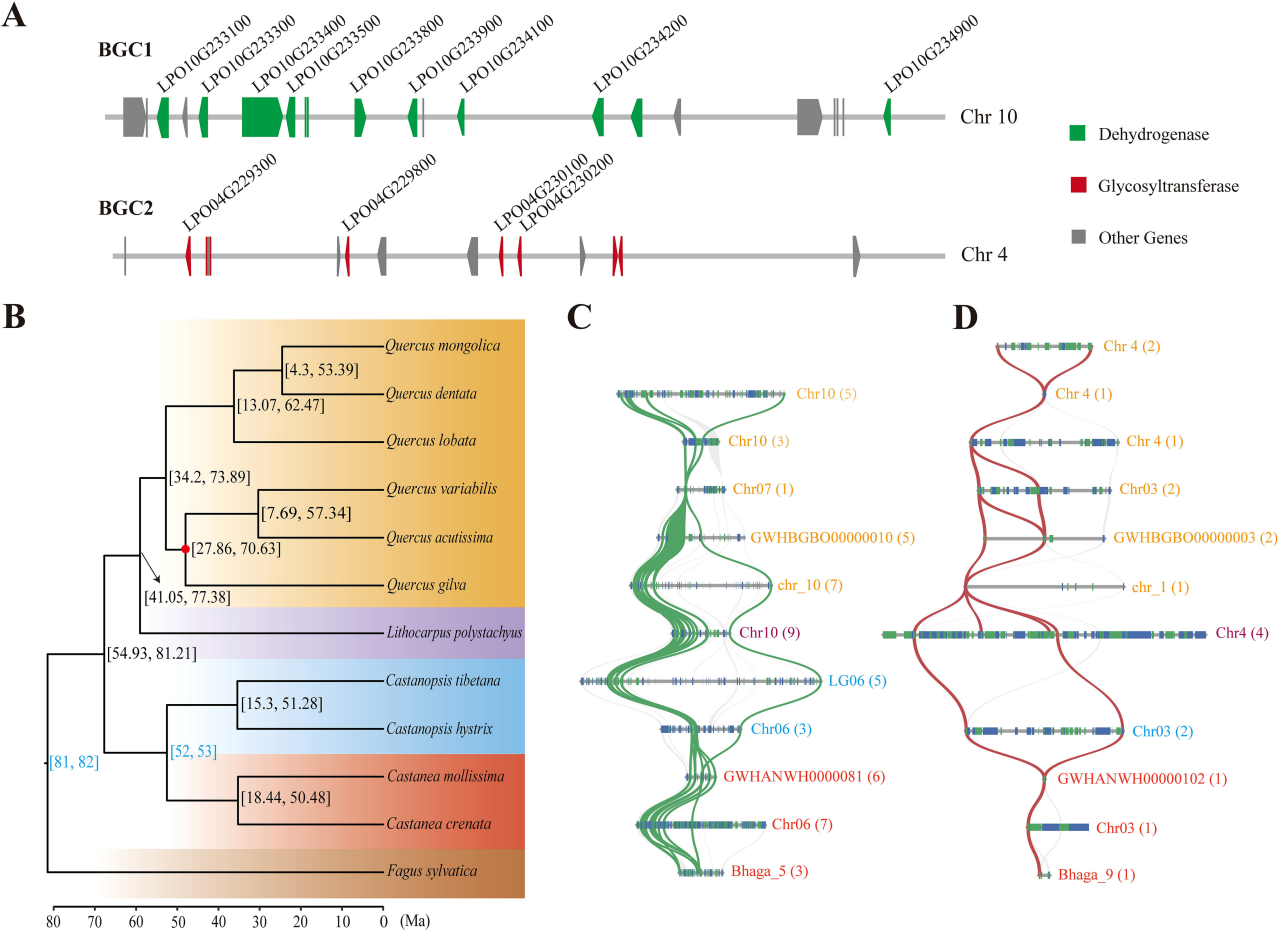
Phylogenomic evolution of Fagaceae species and evolution of the two biosynthesis gene clusters (BGCs). **(A)** BGC1 and BGC2, two biosynthetic gene clusters in the sweet tea genome, including the *CDBR* and *P2’GT* genes respectively. Each dark gray box represents a gene locus, the green and dark red boxes represent genes encoding dehydrogenase and glycosyltransferase respectively, and the candidate genes of *CDBR* and *P2’GT* in DHCs biosynthesis were shown in black words. The arrow of box represents the encoding direction of the gene. **(B)** Phylogeny of sweet tea and 11 other Fagaceae species. The numbers in the square brackets show the 95% confidence intervals of the divergence time. Two fossil calibrations are shown in blue. The red dot at the branch node indicates support lower than 100%. The background color represents different genera of Fagaceae. **(C)** The syntenic genome regions for BGC1 of sweet tea within Fagaceae. **(D)** The syntenic genome regions for BGC2 of sweet tea within Fagaceae. Each blue and green box represents the encoding direction of a gene locus. Syntenic gene pairs of *CDBR* and *P2’GT* were linked by green and red ribbons respectively. The color of pseudochromosome name for each Fagaceae species corresponds to the background color of phylogenomic tree, and the number of syntenic genes of other Fagaceae with sweet tea BGC1 and BGC2 was shown in brackets.

The BGC-detecting tool plantiSMASH was used to identify potential BGCs involved in DHCs biosynthesis. A total of 61 putative BGCs were identified in the sweet tea genome, including eight BGCs that comprise 19 genes involved in the biosynthesis of DHCs. Notably, six of these eight BGCs contain only a single gene (**Supplementary Table S2**). Among the identified clusters, two BGCs (BGC1 and BGC2) were predicted to contain more than three genes associated with DHC biosynthesis. The BGC1 cluster, located on chromosome 10, consists of 21 genes, including 12 that encode dehydrogenase and nine undefined genes. Of the 12 alcohol-dehydrogenase-encoding genes, nine are *CDBR* genes, which are critical for DHC biosynthesis (**Figure 4A**). This cluster is most likely to be involved in essential metabolic processes relevant to DHC synthesis (**Supplementary Table S2**). The BGC2 cluster, located on chromosome 4, is thought to be involved in the synthesis of saccharide-terpene compounds (**Supplementary Table S2**). It contains 15 genes, with eight encoding glycosyltransferases and seven encoding other types of proteins. Significantly, four of the eight glycosyltransferase-encoding genes are *P2’GT* genes in DHCs biosynthesis (**Figure 4A**). Moreover, the nine *CDBRs* and four *P2’GTs* are unevenly distributed across these two BGCs. They are not arranged contiguously, but are instead each associated with one of the respective BGCs (**Figure 4A**).

### 3.5 Phylogenetic relationship and synteny of two BGCs across Fagaceae species

The phylogenetic analysis revealed that sweet tea has the closest relationship with the genus *Quercus*, diverging from *Quercus* at ~59 Ma, with a 95% highest posterior density (HPD) interval of 41.05−77.38 Ma (**Figure 4B**). Syntenic patterns exhibiting a 1:1 correspondence were observed between sweet tea and 11 other Fagaceae species (**Supplementary Figure S3**). Two BGCs show conserved synteny across various Fagaceae species. However, *Quercus mongolica* lacks genes syntenic to BGC1, while *Castanopsis tibetana* lacks genes syntenic to BGC2 (**Figures 4C, D**). At the genomic loci harboring syntenic genes of BGC1 in *Q. dentata* and *Q. variabilis*, uncharacterized BGCs are present (**Supplementary Figure S4**). In contrast, these BGCs are absent in the corresponding genomic regions of other species. Furthermore, the number of syntenic genes identified in other Fagaceae species is lower than the counts of *CDBR* and *P2’GT* genes present within the two BGCs of sweet tea. In particular, only one or two syntenic genes of BGC2 were found in other Fagaceae species, suggesting a potential reduction in the genetic content related to DHC biosynthesis in these species (**Figures 4C, D**).

## 4 Discussion

### 4.1 Accumulation patterns of DHCs in sweet tea leaves

Phlorizin, trilobatin, and sieboldin are three typical sweeteners in the leaves of sweet tea (Yang et al., 1991; Liao et al., 2003). The content of DHCs in leaves is significantly affected by the maturation process (Wang et al., 2022). Therefore, we examined the variation in DHC levels across five distinct developmental stages, from tender to mature leaves. Our analysis revealed an accumulation pattern for phlorizin and trilobatin, with the highest level at stage S3, followed by a sharp decrease at stages S4 and S5. In contrast, sieboldin levels remained high starting from stage S2 onward. The decline in phlorizin and trilobatin levels mirrors the variation of DHC concentrations observed in apple foliage (Hunter and Hull, 1993). However, phloridzin content increases consistently with leaf maturity in sweet tea from Sichuan province (He et al., 2012). The contents of DHCs are influenced by multiple factors, including environmental conditions (Yang et al., 2021; Wang et al., 2022). A decrease in latitude, accompanied by an increase in mean annual temperature and precipitation, positively impacts the growth and development of the sweet tea tree (Huang et al., 2018, 2019). In apple leaves, the deficiency of phlorizin will lead to the narrowing of leaves and the weakening of both the photosynthetic capacity and resistance (Zhou et al., 2019). Compared to Sichuan province, the tropical climate of Guangdong province, characterized by high temperatures and abundant precipitation, accelerates plant leaf growth, leading to rapid metabolism and increased phlorizin consumption, ultimately reducing its accumulation in this region. This finding suggests that, in Guangdong province, the optimal harvest time for phlorizin and trilobatin is approximately 20 days after the emergence of leaf bud. In contrast, it is recommended to collect leaves at the S5 stage to achieve optimal yields of sieboldin, as the content of sieboldin continuously increases with the growth of leaves.

### 4.2 Candidate genes and TFs involved in DHCs biosynthesis

Based on the nine well-characterized enzymes involved in the complete DHC biosynthetic pathway (Gosch et al., 2009; Yahyaa et al., 2016; Gutierrez et al., 2018; Wang et al., 2022), we used genomic data to identify potential candidate genes in sweet tea. Additionally, through Pearson correlation analysis, we pinpointed key genes that were positively correlated with higher concentrations of DHCs in sweet tea, including two *PALs*, two *C4Hs*, 10 *4CLs*, eight *CDBRs*, three *CHSs*, three *F3’Hs*, three *CH3Hs*, one *P2’GT*, and five *P4’GTs*. These genes are hypothesized to play roles in the DHC biosynthetic pathway, though their specific functional roles remain to be experimentally validated. For example, *PAL* is the rate-limiting enzyme in the flavonoids pathways, supplying precursors for downstream metabolites (Dong and Lin, 2021; Zhao et al., 2021). Our analysis revealed that two *PAL* genes were positively correlated with the DHCs accumulation, which might suggest that these two genes play a crucial role at the initiation of the DHC biosynthetic pathway. Moreover, *CDBR* catalyzes the first committed step in DHC biosynthesis, converting 4-coumaryl-CoA to dihydro-4-coumaryl-CoA (Ibdah et al., 2014). Our study identified 10 *CDBR* genes associated with DHC synthesis, highlighting their crucial role in phloretin precursor biosynthesis. While we identified 14 candidate genes encoding *P2’GT*, only one has been confirmed as essential for the biosynthesis of phlorizin. Additionally, we identified five *P4’GT* genes, which are tentatively proposed to be involved in the step prior to the synthesis of trilobatin and sieboldin; however, their specific functional roles and the exact pathway they contribute to remain underexplored and require in-depth functional validation to clarify, particularly to identify whether any of these *P4’GT* genes exert a dominant influence during the synthesis of trilobatin and sieboldin.

The biosynthesis of DHCs involves not only genes encoding enzymes but also transcription factors (TFs) that regulate these processes. In our study, 13 core genes involved in DHC biosynthesis were found to be regulated; our co-expression network analysis revealed strong correlation between TFs such as *MYB*, *WD40*-like, *WRKY*, and *bHLH* with the core biosynthesis genes. *MYB* and *bHLH* are widely distributed in plants, and it has been confirmed that they play a crucial regulatory role in the biosynthesis of flavonoids and phenolic acids (Xu et al., 2015; Pratyusha and Sarada, 2022). Notably, two transcription factors containing *MYB*-like DNA-binding domains have been shown to regulate DHC glycoside patterns in various apple tissues (Wang et al., 2023). Additionally, *MYB* frequently interacts with *bHLH* and *WD40* to initiate the transcription of specific genes (Pratyusha and Sarada, 2022). Therefore, the TFs identified in our study, along with the candidate DHC biosynthesis genes, represent potential targets for further functional studies in sweet tea.

Genes duplication has played a crucial role in the diversification of metabolites (Leong and Last, 2017; Louveau and Osbourn, 2019; Hansen et al., 2021). In our study, TD or PD increased the copy number of genes encoding *PAL*, *4CL*, *CDBR*, *CH3H*, *P2’GT*, and *P4’GT* enzymes. Our findings suggest that the biosynthesis of trilobatin and sieboldin is regulated by five tandem-arranged *P4’GT* genes (*LPO02G147400.1*, *LPO06G039500.1*, *LPO06G039600.1*, *LPO06G039800.1*, and *LPO09G074900.1*). Additionally, two *4CLs*, four *CDBRs*, and three *CH3Hs* were formed through tandem duplication. This genetic pattern strongly suggests that TD may serve as a regulatory mechanism in the DHC biosynthetic pathway. Yet, comprehensive experimental studies are needed to clarify and validate the exact role of TD in this pathway.

### 4.3 The BGCs in DHCs biosynthesis of sweet tea

There have been no previous reports regarding the BGCs involved in the biosynthesis of DHCs in plants. In this study, we report two BGCs that include candidate *CDBR* and *P2’GT* genes, which may catalyse the synthesis of precursor of phloretin, and phlorizin, respectively. Phylogenetic and syntenic analyses of BGC1 in Fagaceae family reveal an ancient gene cluster inherited from a common ancestor, co-inherited by several *Quercus* and *Lithocarpus* species, indicating their shared evolutionary heritage. In contrast, BGC2 is found exclusively in sweet tea, strongly suggesting that it likely originated independently in Fagaceae, though this remains to be supported by direct experimental evidence. Compelling evidence indicates that the plant BGCs reported so far have originated through processes such as gene duplication, relocation, and neofunctionalization (Nützmann et al., 2016). BGC1 contains nine candidate genes encoding *CDBR* enzyme, eight of which originated from gene duplication, while the uncharacterized BGC of *Q. variabilis* contains only one *CDBR* gene. The synteny observed between BGC1 and the uncharacterized BGC of *Q. variabilis*, supports the hypothesis that an original founder gene of *CDBR* first acquired additional genes, with subsequent gene duplication events leading to the establishment of the BGC1 architecture. BGC2 is composed of four *P2’GT* genes, two of which were produced through gene duplication; however, the specific formation process remains unclear.

Determining the association between a predicted BGC and its metabolites remains a significant challenge. BGC1 contains nine *CDBR* genes, four of which are crucial for the regulation of the phlorizin precursor substances. Among them, one is regulated by a transcription factor. While only one *P2’GT* gene is involved in the synthesis of phlorizin, this gene is absent in BGC2. Therefore, the specific functions of this gene cluster still require further exploration.

## 5 Conclusion

The tender leaves of sweet tea provide an ideal resource for the extraction and utilization of DHCs. Metabolomic analysis revealed that four DHCs reached their highest contents approximately 20 days after the emergence of leaf buds, while sieboldin exhibited a high level. By integrating genomic, transcriptomic, and metabolomic data, we reconstructed the biosynthetic pathway for DHCs and identified 82 enzyme-encoding genes. Two *PALs*, eight *CDBRs*, three *CHSs*, one *P2’TG*, and five *P4’GTs* genes play pivotal roles in regulating DHC biosynthesis. Furthermore, TD or PD promotes the assembly of these genes. Co-expression network analysis revealed that four modules were positively correlated with DHC levels, suggesting that *MYB*, *WD40*-like, *WRKY*, and *bHLH* TFs may regulate the biosynthesis. Two BGCs were characterized using genome mining, and BGC1 may have evolved from an ancient gene cluster in the Fagaceae family. In contrast, BGC2 emerged concurrently with the formation of *L. litseifolius*. The genomic, transcriptomic, and metabolomic resources, along with the experimental results presented in this study, will promote the efficient utilization of sweet tea as a herbal medicine and facilitate further functional genomic studies in the future.

## Data Availability

The datasets presented in this study can be found in online repositories. The names of the repository/repositories and accession number(s) can be found in the article/**Supplementary Material**.
