# Supplementary material for: Integrated metabolomic and transcriptomic analysis reveals the biosynthesis mechanism of dihydrochalcones in sweet tea (Lithocarpus litseifolius)
Source: Front Plant Sci. 2025 Aug 4;16:1629266. doi: 10.3389/fpls.2025.1629266 (PMC12358465; doi:10.3389/fpls.2025.1629266)
Supplement: Supplementary file 1 [file DataSheet1.docx]

Supplementary Figures


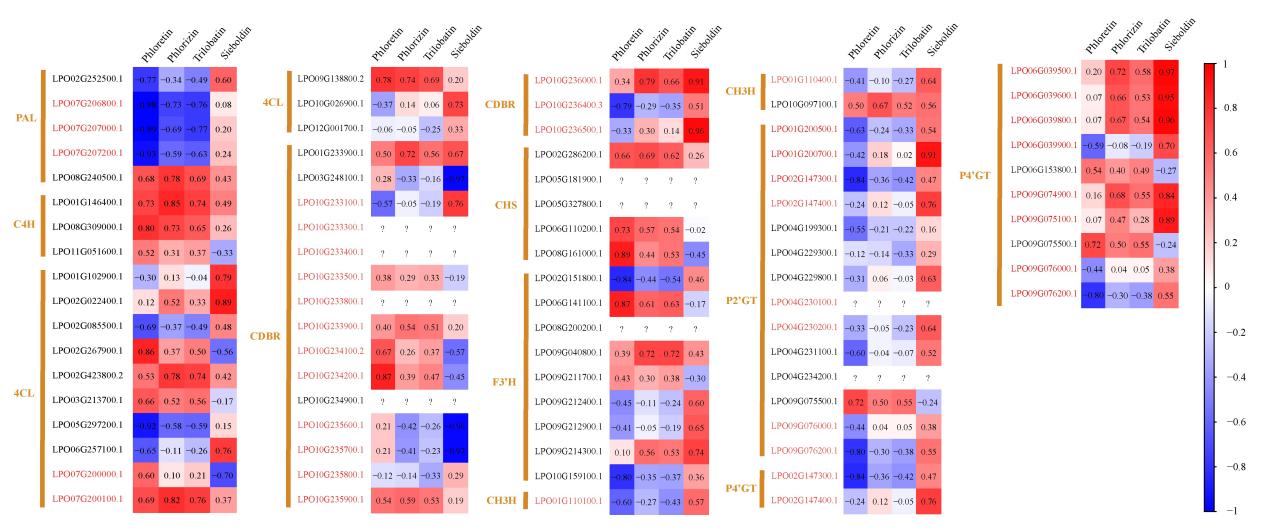


**Supplementary Figure 1.** Pearson correlation analysis of the content of phloretin, phlorizin, trilobatin, and sieboldin with genes of the synthesis pathway.


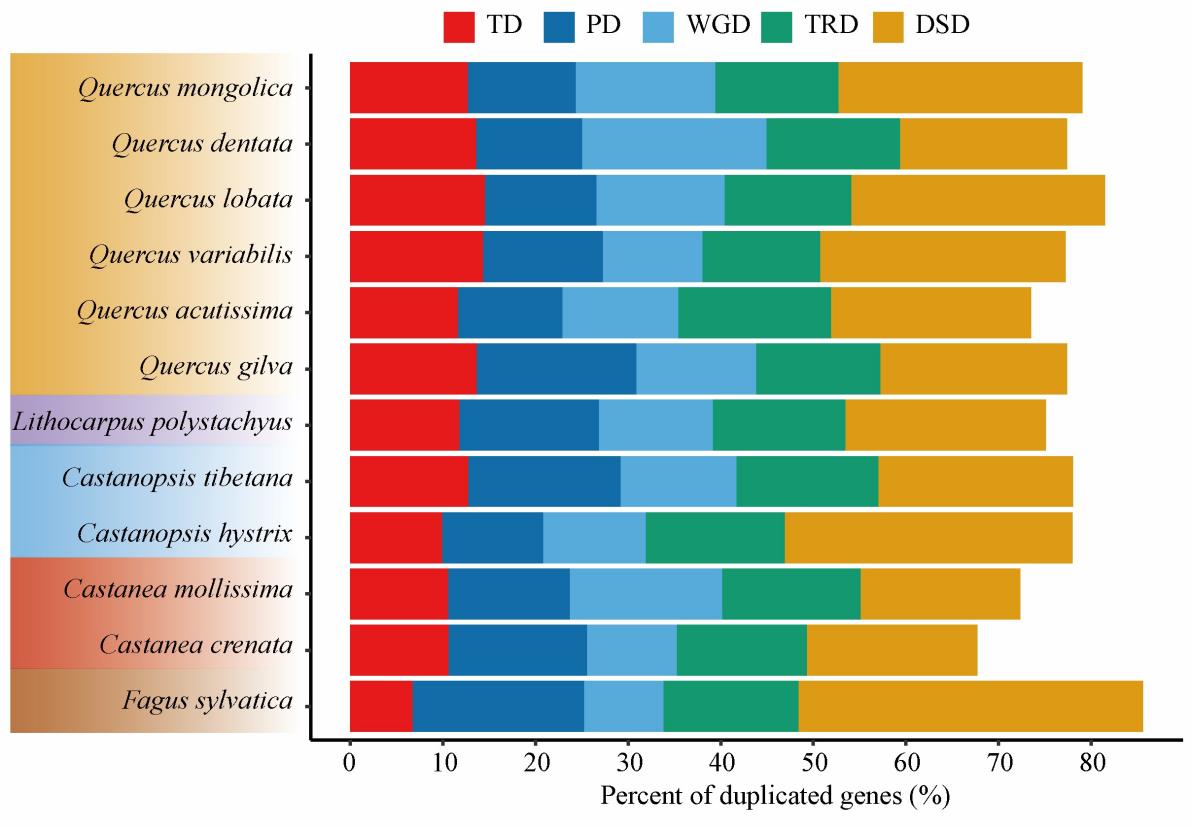


**Supplementary Figure 2.** Percentages of the different types of gene duplications in sweet tea and 11 Fagaceae species.


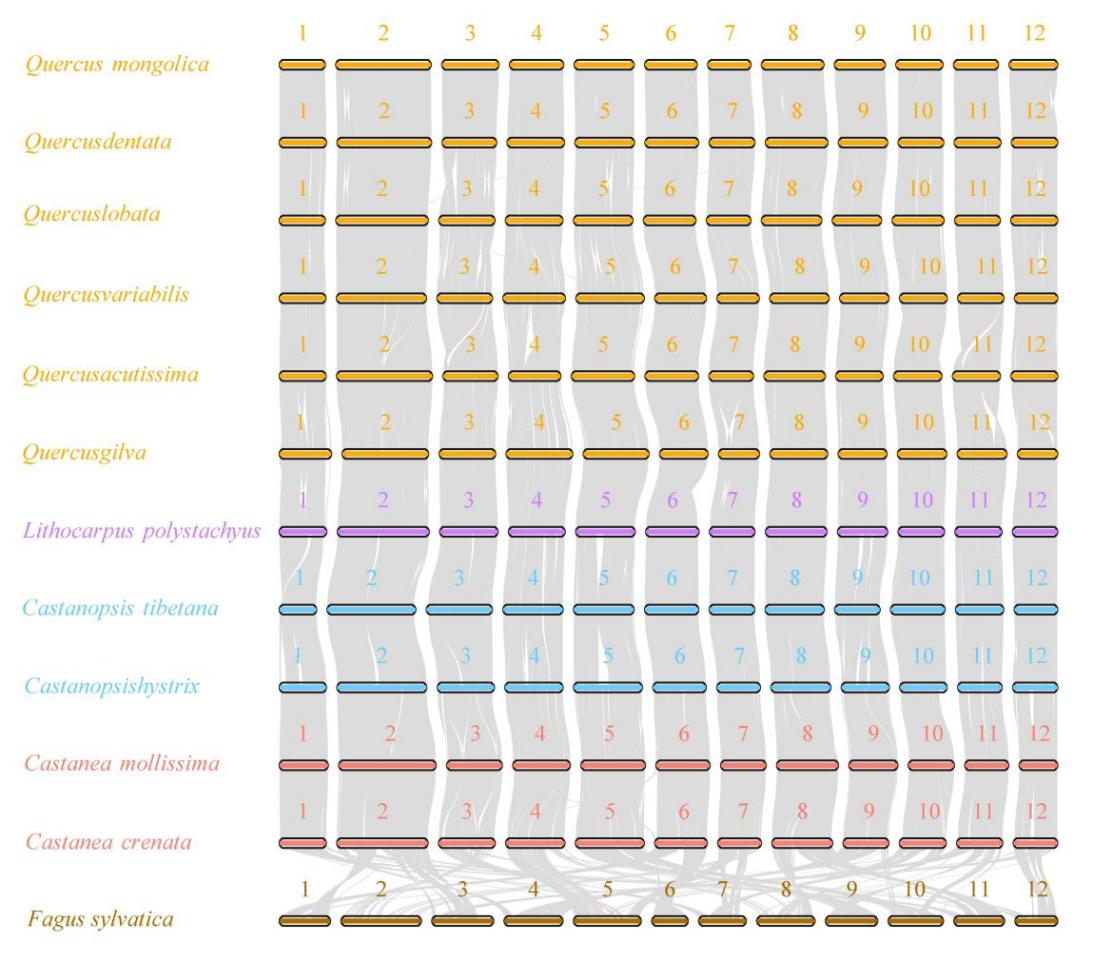


**Supplementary Figure 3.** Syntenic blocks between sweet tea and 11 other Fagaceae species. Syntenic blocks are linked by gray ribbons, and the colour of species name corresponds to different genera of Fagaceae.


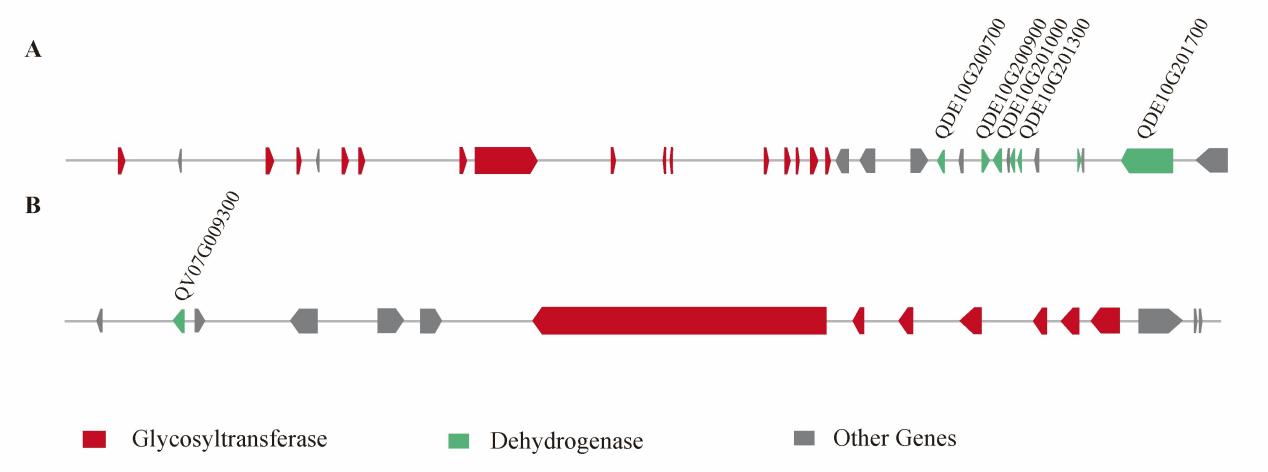


**Supplementary Figure 4.** Two potential biosynthetic gene clusters (BGCs) in the (**A**) *Q. dentata* and (**B**) *Q.variabilis* genome. Each box represents a gene locus, the green and dark red boxes represent genes encoding dehydrogenase and glycosyltransferase respectively, and the syntenic candidate genes of CDBR with sweet tea in DHCs biosynthesis were shown in black words. The arrow of the box represents the encoding direction of the gene.
